# Electrospun Polycaprolactone Nanofibers: Current Research and Applications in Biomedical Application

**DOI:** 10.34172/apb.2022.070

**Published:** 2021-10-03

**Authors:** Arezo Azari, Ali Golchin, Maryam Mahmoodinia Maymand, Fatemeh Mansouri, Abdolreza Ardeshirylajimi

**Affiliations:** ^1^Department of Tissue Engineering and Applied Cell Sciences, School of Advanced Technologies in Medicine, Shahid Beheshti University of Medical Sciences, Tehran, Iran.; ^2^Department of Clinical Biochemistry and Applied Cell Sciences, Faculty of Medicine, Urmia University of Medical Sciences, Urmia, Iran.; ^3^Regenerative Medicine Group (REMED), Universal Scientific Education and Research Network (USERN), Urmia, Iran.; ^4^Cell Therapy Center, Sarem Women’s Hospital, Tehran, Iran.; ^5^Department of Genetics and Immunology, Faculty of Medicine, Urmia University of Medical Sciences, Urmia, Iran.; ^6^Cellular and Molecular Research Center, Urmia University of Medical Sciences, Urmia, Iran.; ^7^Urogenital Stem Cell Research Center, Shahid Beheshti University of Medical Sciences, Tehran, Iran.; ^8^SinaCell Research and Product Center, Tehran, Iran.

**Keywords:** Electrospinning, Regenerative medicine, Polycaprolactone, PCL, Stem cells, Tissue engineering

## Abstract

Unique mechanical properties, miscibility potency, and biodegradability are the three prominent features of polycaprolactone (PCL), making it an attractive biomaterial which commonly applied in regenerative medicine and biomedical engineering. Different strategies developed for fabricating nanofibrous construct, electrospinning is a practical, simple, and efficient technique based on electro-hydrodynamic systems that use an electrified viscous fluid jet drawn by the air toward a collector at a changing electric potential. PCL electrospun-based nanofibrous composites as proper scaffolds are employed in stem cell-related research, particularly in tissue engineering, wound dressing, and systems designed for sending drugs. A compilation of mechanochemical properties and most common biological performance on PCL-based electrospun fibrous structures in biomedical application are included in this study. Therefore, electrospun PCL nanofiber applying has been presented, and after that, current progress and prospects have been discussed. Literature reviews revealed that electrospun PCL nanofibrous composites had gained significant attention in regenerative medicine, and these structures have shown notable development in mechanobiological properties. This evidence is a crucial success for biomedical strategies, especially in regenerative medicine.

## Introduction

Polycaprolactone (PCL) is an aliphatic, semicrystalline, thermoplastic polyester that is soft at room temperature. Two leading pathways to manufacturing PCL have been introduced in the different references, including ring-opening polymerization (ROP) of a ɛ-caprolactone (ɛ-CL) and polycondensation of the 6-hydroxyhexanoic acid.^1^ PCL is a polymer confirmed by the Food and Drug Administration (FDA) extensively studied to be used in diverse biomedical areas, including tissue engineering, systems developed for sending drugs, and implantable biomaterials.^2-4^ Given the polymer and environmental condition and physicochemical properties, biodegradation of PCL was performed within several weeks to several years.^5-7^ Degradation via autocatalysis, enzymes (in the body), and microbes make the biodegradation fate of PCL.^8^ PCL demonstrates the unique feature of being mixable with different polymers, including cellulose,^9^ carbonate polymers,^4^ poly (ethylene oxide), poly (vinyl chloride), poly (styrene-acrylonitrile), poly (acrylonitrile butadiene styrene), poly (bisphenol-A),^10^ poly (3-hydroxy butyrate),^11^ and even biological compounds (such as growth factors, drugs, and natural products^4^). Ordinarily, the PCL-related peaks are displayed utilizing FTIR spectra of the PCL polymer at 1729.6 and 1185.9/cm that demonstrate C─C (═O)─O carbonyl stretching and axial deformation.^4^ PCL established unique properties (Table 1) as a candidate polymer to be used in biomedical engineering and regenerative therapeutics. According to different purposes, several methods are studied to fabricate PCL-based compounds and scaffolds. Several techniques, including electrospinning, force spinning, melt blowing, self-building, and template synthesis, are reported to modify the diameter of the fibers in the range of between micro to nano dimensions. PCL electrospun nanofibrous structure makes one of the well-known constructs of PCL-based composites for application in regenerative medicine.

**Table 1:** The principal properties of PCL

**Properties**	**Range/Description**	**Ref.**
Molecular weight (Mn/g mol^-1^)	3000-80 000	^12^
Density (r/g cm^3^)	1.071-1.200	^13,14^
Tensile strength (σ/MPa)	4-785	^15,16^
Young modulus (E/GPa)	0.21-0.44	^15,16^
Elongation at break (e/%)	20-1000	^15,16^
Crystallinity (%)	69%	^13^
Functional groups and FTIR absorption wavenumbers	Asymmetric -CH_2 _(2943 cm^-1^), symmetric -CH_2_ (2866 cm^-1^), Carbonyl (1722 cm^-1^), C-O, and C-C (1293 cm^-1^), asymmetric C-O-C (1239 cm^-1^)	^17^
Glass transition temperature (T_g_/^º^C)	(-65)-(-60)	^15,16^
Tensile stress at break or max (MPa)	14	^16^
Water permeability at 25^º^C (g/m^2^/day)	177	^16^
Surface tension (g) in mN/m	35.5	^18^
Melting temperature (Tm/^º^C)	56-65	^16^
Decomposition temperature (/^º^C)	350	^15,16^
Inherent viscosity (Zinh/cm^3^ g^-1^)	100-130	^15,16^
Intrinsic viscosity (Z/cm^3^ g^-1^)	0.9	^13^
Solubility	Highly soluble: Chloroform, dichloromethane, carbon tetrachloride, toluene, cyclohexanone, 2-nitropropane; cyclohexanone, pyridine, dichlorobenzene, teraline, toluene, styrene, cyclobenzene	^9,16,19^
	Partially soluble: acetone, 2-butanone, ethyl acetate, dimethylformamide and acetonitrile, o-dichloroethane, tetrahydrofuran, acetophenone	
	**Insoluble:** water, alcohol, petroleum ether, diethyl ether, acetonitrile, nitromethane, dimethyl sulfoxide, di(ethylene glycol), di(propylene glycol), tetrachloroethylene, glycerol, methanol, ethanediol, ethanol, propylene glycol, 2-ethyl hexanol, cyclohexanol, diethyl carbonate, cyclohexane, carbon disulfide	

Electrospinning is a fiber production technique that practices electric force to form charged threads of polymer solutions into fibers in nano-micro scale diameter. In other words, electrospinning is a fabrication device that can be applied electrostatic processing to manufacture fibrous polymer mats containing various fiber diameters ranging.^20,21^ Electrospinning was patented as a method to process polymers with well-regulated average size, porosity, and morphology, for creating resultant scaffolds, which has been the subject of intensive research.^15,22^ A key advantage of electrospinning is the complete spectrum of well-controlled parameters. These parameters are redivided into three significant categories: solution parameters, processing parameters, and ambient parameters (Table 2).^23^ However, we do not want to discuss the electrospinning process. Up to date, electrospun nanofibers are produced using more than 100 various polymers through synthesizing and organic products. The polymeric nanofibers are produced using an either solvent or melt spinning. In addition, electrospinning can prepare different structures such as core-shell, multilayers, and/or blended, based on the ideas of studies.^20^ Hence, an electrospun nanofiber acts as a valuable option in drug and cell delivery systems and would be a more usual approach that could considerably bypass some of the challenges and limitations.

**Table 2:** Effective parameters in the electrospinning process

**Parameter**	**Effects**
Applied voltage	↓ – larger fibers, ↑ – smaller fibers
Flow rate	↓ – smaller fibers, ↑ – larger fibers
Collector distance	↓ – smaller fibers, ↑ – larger fibers
Needle diameter	↑ needle diameter – larger fibers
Solution viscosity	↓ – bead formation, ↑ – larger fibers
Solution conductivity	↑ – uniform bead-free fibers, smaller fibers
Humidity and temperature	↑ temperature – ↓ viscosity, ↑ humidity –appearance of circular pores on the fibers

As mentioned above, one of the common PCL-derived structures that recently has attracted the attention of biomedical researchers in regenerative medicine programs is electrospun PCL nanofibers. On the other hand, literature review and reports demonstrate that the electrospinning of PCL with other polymers and electrospun fibers begets more potent advantages over the pure electrospun PCL. For instance, human β-nerve growth factor contained electrospun nanofibers were developed by encapsulating the two polymers of PCL and ethyl ethylene phosphate, and nanofibers’ biological behavior and neuronal differentiation range were assessed by seeding rat pheochromocytoma cells over nanofibers for up to three months.^10^

**Figure 1:**
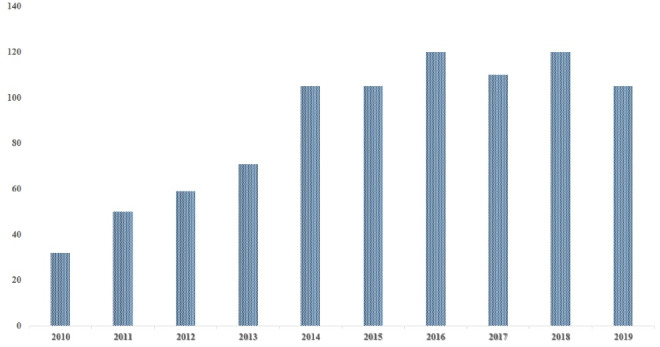
Number of published scientific papers related to “electrospinning” and “PCL” according to NCBI (MEDLINE) from 2010 to 2019; date assessed, 20 July 2020

**Figure 2:**
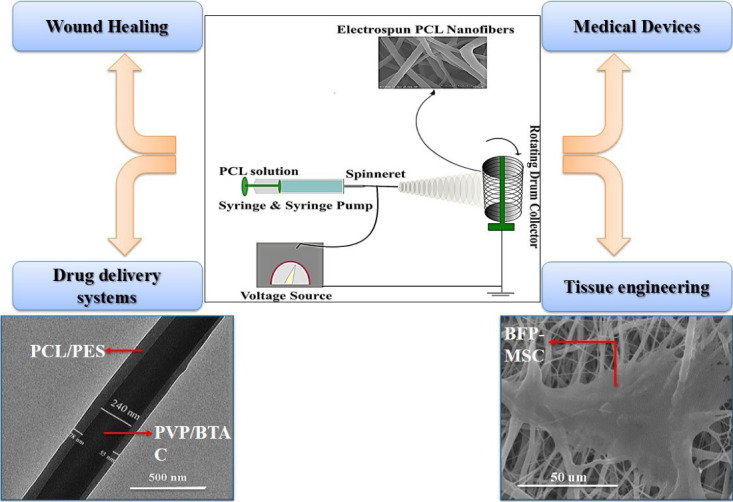
Schematic illustration of electrospun PCL-based nanofibrous constructs usage in biomedical applications. SEM images were used from^24-26^ (Abbreviation: Poly (ethylene succinate) (PES), poly(vinylpyrrolidone) (PVP), enzyl dimethyl tetradecyl ammonium chloride (BTAC), Buccal fat pad derived mesenchymal stem cell (BFP-MSC))

The development of PCL nanofibrous composites by employing electrospinning-based techniques to provide a smooth surface using a sizeable surface-area-to-volume ratio and other appropriate characteristics have been well investigated during the last two decades. Hence, several studies have investigated producing various electrospun PCL nanofibrous composites to evaluate those potentials in regenerative medicine for either vivo or in vitro research. The annual number of studies on “electrospinning” and “PCL” during the past decade which have been indexed in MEDLINE is provided in Figure 1. Applications of electrospun PCL-based structures are included regenerative medicine branches including tissue engineering, bandaging wounds, and drug delivery systems. Figure 2 represents applications of electrospun PCL-based nanofibers in different fields of the biomedical area.

 In the present study, the current developments of electrospun PCL nanofibers in regenerative medicine branches are investigated. Electrospun PCL nanofibers applications have been presented, and recent progress and prospects are discussed. According to the literature review and contributed content, this mentioned structure has obtained a great interest in the regenerative medicine field, so this paper is advised to researchers and those interested in this field.

## PCL and combined polymers

**Figure 3:**
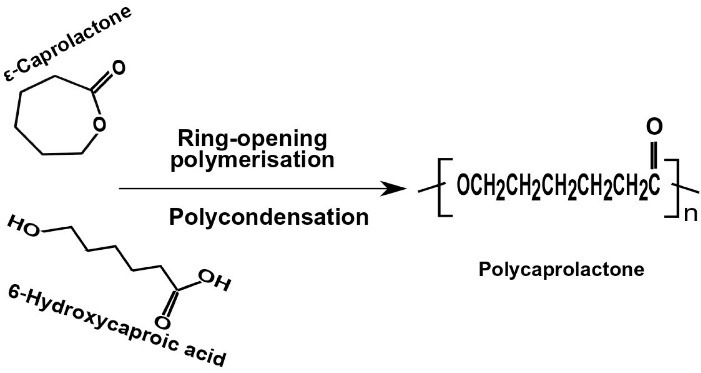
Synthesis of PCL by two common pathways

PCL is one of the biodegradable and biocompatible materials among other polymers, which have widespread utilization in biomedical science. There are two pathways for PCL synthesis; a ROP, which can be catalyzed by ionic initiators, metal carboxylate, and alkoxides (at temperature > 120ºC).^27^ The other method is the polycondensation of 6-hydroxyhexanoic acid; however, this method has limitations, including the low quality of products^28^ (Figure 3). It has good rheological properties also; low melting temperature (glass transition (T_g_) ≈60 ºC, melting temperature (T_m_) ≈60ºC). The disintegration of PCL depends on the chemical structure, including molecular weight (M_n_) and end group chemistry, which displays remarkable and fast thermal degradation when temperature blows over 170ºC. Furthermore, the ester group on PCL means to degrade through hydrolysis from chemical and enzymatic pathways.^14,29^

As we know, PCL miscibility potency makes one of the primary support of this polymer for its clinical usage. Hence, PCL copolymers have widely been used in basic and clinical investigations. For instance, PEG–PCL copolymer is a proper biomaterial that has steadily been utilized for therapeutic purposes.^30–32^ As another example, a blended PCL with polypropylene or polyethylene and spun into fibers develop a notably efficient pigment dispersing aid.^33^ Recently, PCL electrospun membrane combined with bioglass was introduced as a promising biomaterial to promote bone tissue regeneration.^34^ In this study, the physical and chemical analysis results confirmed progress in fabricating bioactive electrospun membranes that significantly increased cell viability and osteoblast differentiation.^34^ Generally, PCL-based electrospun scaffolds constructed from other aliphatic polyesters (PE)-based copolymers, such as polylactic-co-glycolic acid (PLGA), polyglycerol sebacate (PGS), poly(ethylene adipate) (PEA), and polylactic acid (PLA), are trialed out for tissue-engineered scaffolds applications. However, target tissues and defined objectives determine the type of chosen copolymers. In the latest study, the structure, composition, and properties of three most commonly accepted polyester-based biopolymer materials combined with PCL at 2:1 (wt.%) ratio, including PGS/PCL, PLGA/PCL, PLLA)/PCL and pure PCL as carrier vehicles, were investigated for retinal progenitor cell (RPC) attachment and RPC proliferation, and finally reported that PGS/PCL scaffolds improve RPC attachment and RPC proliferation more favorably compared to other polymeric blends and pure PCL.^35^ However, copolymers are crucial to the medical industry as they present the means to combine properties of different materials formulating new unique biomaterials.

## Effect of solvents on PCL and combined polymers

It should be considered, the type of solvent is one of the critical parameters in the morphology of PCL and combined polymers; other factors related to solvents in the electrospinning technique are the concentration and ratio of used solvent and polymer. For investigating the solubility of PCL at room temperature in various solvents, can utilize, for instance, Chloroform, toluene, benzene, carbon tetrachloride, cyclohexanone, tetrahydrofuran (THF), 2-nitropropane, dimethyl carbonate (DMC), and dioxane dichloromethane (DCM). This polymer partially soluble in numerous solvents, including dimethylformamide (DMF), ethyl acetate, acetone, acetonitrile, and 2-butanone,^15^ is entirely insoluble in petroleum ether, diethyl ether, alcohol and, water. The PCL may be miscible with some polymers, including poly(styrene-acrylonitrile), poly(bisphenol-A), poly (vinyl chloride), and poly (acrylonitrile butadiene styrene); from a mechanical aspect, it is compatible with some of the polymers, for instance, poly-(vinyl acetate), polyethylene, polypropylene, etc.^9,14,29,36–38^

Maccaferri et al investigated the solubility parameter $\left(\bar{\Delta \delta }\right)$ for NBR/ PCL; this parameter was obtained 7.9MPa^1/2^ (if the solubility difference of two pair of polymer is a small value, $\bar{\Delta \delta }$ < 5MPa^1/2^ good miscibility occurs, and partial miscibility may be received potentially up to 10MPa^1/2^). DMAc was the solvent that tested for NBR electrospinning, but it did not have a suitable outcome for PCL. They successfully prepared a 10%wt PCL solution in CHCl_3_/DMF 1:1wt mixture (SPCL) and homogeneously blended it with S-NBR. The polymer ratio is critical in this work. Different polymer ratios were utilized to evaluate the threshold of polymer amount that causes the separation or precipitation of components in the complex mixtures. As regards, there were no such events observed, and the blend solution was clear. It was apparent to follow a drop in the diameter of the fibers when the PCL was in the blend; it could happen due to various polymer/solvent system mixtures which applied for all electrospun sample. Because of the NBR presence, this kind of scaffold could show low-temperature Tg, and interestingly, high PCL-like crystal phase content was boosted. It should be noticed; the morphology could compare with thermoplastic elastomers and achieved remarkable long-term stability with no need for chemical rubber cross-linking in this research.^39^

Silva et al fabricated a kind of coaxial electrospun scaffold in which the core was poly (glycerol sebacate) (PGS) - Kartogenin (KGN) and the shell part was PCL. The solvent system includes 2, 2,2-trifluoroethanol (TFE): a mixture of DMSO: TFE (volume ratio 20:80) for PCL and PGS, KGN. In this work, the different morphological types with average fiber diameters (505-738 nm) were investigated, including non-aligned PCL (593 ± 248 nm), aligned PCL (505 ± 220 nm), non-aligned coaxial PGS/PCL (738 ± 274 nm), and aligned coaxial PGS/PCL (617 ± 235 nm) nanofibers. According to the SEM results, the electrospun scaffolds had high porosity and interconnectedness. These proprieties of the scaffolds provided a sufficient surface area for cell adhesion due to media and oxygen diffused through the scaffold quickly. The elastic modulus for coaxial PGS/PCL aligned was nearly 11.8 ± 0.7 MPa this value is a little higher but near to findings of previous research on PGS-PCL mix aligned.^40^

Chiesa and colleagues conducted preparing PLA-PCL/GNPs (Graphene nanoplatelets) electrospun mats for biomedical applications. The solvent considered for dissolved PLA-PCL was methylene chloride (MC); final concentration was obtained 28.5% w/v. GNPs were homogeneously stringed in DMF and mixed the polymeric mixture, and the proportion of MC: DMF was considered as 70:30. The final polymer (PLA-PCL) concentration was 20%w/v in the solvent mixture. Rheological property is the key parameter for achieving homogeneous electrospun matrices with constant fibers. According to the viscosity, the resistance to flow would change; they have a direct relationship whenever viscosity is high, the resistance of flow will arise, and the diameter of the nanofiber will increase as the viscosity increase. Due to the reported paper, the values represented remarkable enhancement from 0.63 Pa.s to 1.25 Pa.s, 1.55 Pa.s, 1.93 Pa.s, and 3.02 Pa.s for PLA-PCL, blend GNPs-0.5%, GNPs- 1wt%, GNPs-2wt% and GNPs-4wt%, respectively. Then, when GNPs added up to 4wt% to PLA-PCL solution, the dynamic viscosity was five times greater than before. About the morphology of the fiber, which presented homogeneous fibrous matrices with the 1µm diameter of the fiber and the porosity is suitable according to the tested analysis.^41^ In Table 3, the other recent research has shown and the specific summary of the morphology of the blend polymer.

**Table 3:** Summary of some of the PCL-based electrospinning related studies

**No**	**Combined polymers**	**Solvent system**	**Biomedical application**	**Morphology of combined polymers**	**Ref**
1	PCL/PLGA/TFV	**PCL/PLGA**: hexafluoroisopropanol (HFIP), 15% (w/v) **TFV:** polymer solutions at 10– 40% (w/w)	Drug release (Tenofovir (TFV))	Fibers without TFV showed smooth and no defect on morphology, but with 15 wt% of TFV, the surface aggregation displayed. The inhomogeneities decreased by PLGA content in fiber morphology. Fiber diameter for PCL 2.0 ± 0.3 μm and 1.1 ± 0.1 μm for PLGA were obtained	^42^
2	PLA-PCL/GNPs	**PCL**: MC **PLA**: MC **GNPs**: DMF MC:DMF 70:30, 20% w/v	-	GNPs concentration prominently influenced the fibers morphology and diameters distribution, mobility of PLA–PCL chain in the crystallization process. It could able to tune the mechanical and thermal features of the electrospun matrices.	^41^
3	Col-c-PCL TiO2-i-PCL	**PCL**: chloroform/methanol mixture,3:1 v/v **Col**: TFE(80 mg/ml)	Skin tissue engineering	The fiber diameter affects by PCL concentration and electrostatic repulsion force. It could decrease when the PCL concentration is 11 and13%, with voltage increasing. At the lower voltage, about 10 kV, the ribbon–shape of the nanofiber was observed. At the high voltage range, continuous nanofibers with 2.0 to 0.4 μm diameter distribution were achieved.	^43^
4	PCL/PLA	**PCL/PLA(4/1)**: DCM/DMF(60/40) overall concentrations: 8 wt%	Stem cells osteogenic differentiation, Cranial bone formation	The thermally-induced nanofiber self-agglomeration (TISA) technique was used for fabricating PCL/PLA-3D nanofibrous scaffolds. The nanofiber diameter ranging was from 200nm to 1 μm, 150nm to 2 μm for PCL and PCL/PLA, respectively.	^44^
5	pNSR32/PCL/Gt (Recombinant spider silk protein (pNSR32) gelatin (Gt))	**PNSR32/PCL/Gt**: Formic acid (98%)	Small Caliber Vascular tissue engineering	Three categories were considered in this work, including pNSR32/PCL/Gt, PCL, pNSR32/PCL scaffolds; the average diameter was 171 ± 23 nm, 116 ± 30 nm, 112 ± 23 nm, respectively. In the presence of pNSR32, the PCL fiber diameter did not change. Smooth surface, randomly oriented, interconnected pore structure appeared in all scaffolds.	^45^
6	NBR/PCL	**PCL**: CHCl_3_/DMF,1:1wt; S-PCL, 10%wt; (1.0 g of polymer in 3.0 mL of CHCl3 and 4.8 mL of DMF) **NBR**: DMAc; S-NBR, 10%wt (1.0g of polymer in 9.6mL of solvent)	-	The diameter of the blend fiber is significantly smaller than N-PCL (nanofiber PCL). Mechanical performances were raised due to the morphological character has been improved.	^39^
7	PCL/ PGS (incorporating silicate, borosilicate bioactive glass (BG))	**PCL**: acetic acid (20% w/v) (PGS was added to the solution)	Soft tissue engineering	The addition of PGS caused an increase of the average fiber diameter, which PCL average fiber diameter 0.9 ± 0.4 µm falls within the range of 0.11–3.85 µm. But the presence of BG particles showed an increase in the fiber diameter distribution, with no change in average fiber diameter significantly.	^46^
8	PCL/COL-HA	**PCL**: mixture of Chloroform and acetic acid, 50:50(10% (w/v)) **Col**: 0.2 N acetic acid **HA**: 0.8 M sodium chloride	peripheral nerve regeneration	Electrospun PCL fibrous mat was rolled within a polystyrene cylindrical mold with a diameter 6 mm and filled by Col-HA blend solution. HA can effect on mechanical properties, sponge porosity, degradation rate, and water absorption. Good adhesion occurred between PCL and Col-HA, and the fibrous showed a similarity to the rat sciatic nerve in terms of mechanical properties.	^47^
9	PGS(core)- KGN /PCL(shell)	**PCL**: TFE, 10% w/v (shell solution) **PGS**: TFE, 80% w/v (core solution) **KGN**: mixture of DMSO: TFE (volume ratio 20:80	Cartilage tissue engineering	The core-shell arrangement of the coaxial fibers displayed a significantly lower effect on the electrospun scaffold’s elastic modulus than fiber alignment.	^40^
10	PCL/ Gel	**PCL/Gel **50/50 (w/w): TFEA and TCM (2/3, v/v), 7% (w/v) Trichloromethane (TCM), 2,2,2-Trifluoroethanol (TFEA)	Tendon repair	Random and aligned nanofibers with 425.28 ± 48.15 nm, 427.82 ± 56.99 nm diameters, respectively, were fabricated.	^48^
11	PCL/Carbomer	**PCL: **DMF/chloroform (1:9)**Carbomer:** DMF	Wound healing	Fibers were in random orientation and bead-free with interconnected pores. Fiber diameters of was 1378 ± 259.82 nm.	^2,4^
12	PCL/Magnesium Oxide	DMF/chloroform	Bone tissue reconstruction	The diameter of nanofibers significantly decreased from 1029.25 ± 209.349 μm to 537.83 + 0.140 nm.	^49^

## Electrospun PCL nanofibers in biomedical application

###  Electrospun PCL nanofibers and tissue engineering

**Figure 4:**
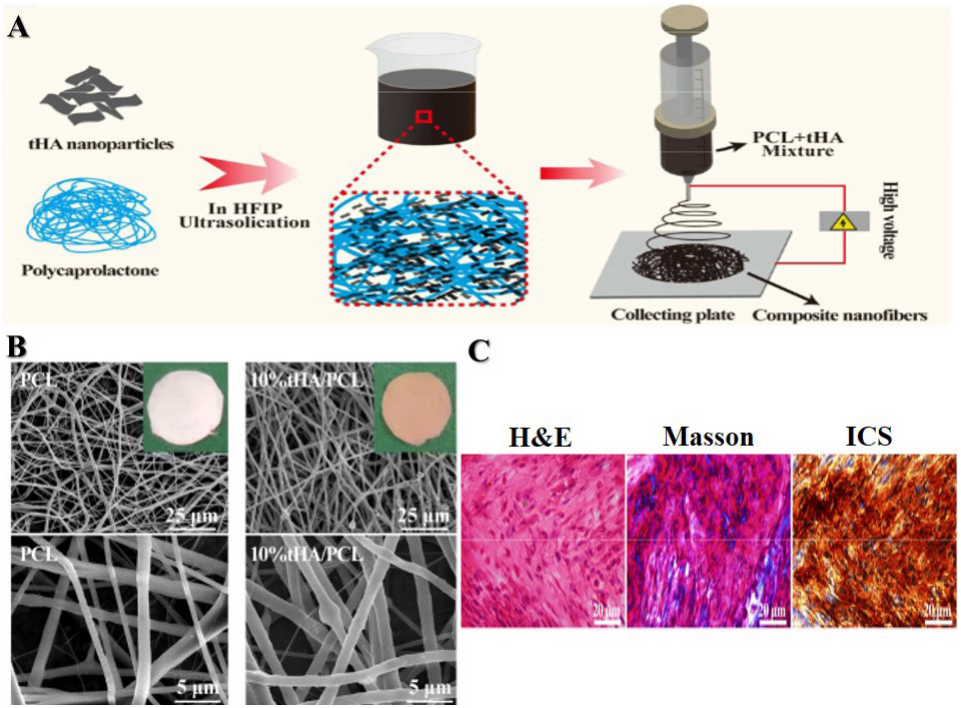
An example of an electrospun PCL-based nanofibrous scaffold in medical research. (A) Schematic illustration of the process to fabricate tHA/PCL composite nanofibers using the electrospinning technique. (B) SEM images of Blank PCL and tHA/PCL nanofibrous scaffolds. (C) Elected histological sections of human mesenchymal stem cells -10%tHA/PCL constructs implanted subcutaneously in mice dorsa after 4 weeks. Reprinted with permission from Gao et al,^57^ American Chemical Society (ACS)

To biologically control the behavior of the cells from separate sources in tissue engineering, scaffolds are generally developed to simulate chemical, physical and natural conditions of the fundamental stem cell niches. MSCs are one of the main stem cell sources currently used in cell-based therapy^50,51^ and tissue engineering repeatedly.^49^ Scaffolds based on PCL systems could be provided a functional microenvironment surface with an elevated surface area similar to the native tissue while assimilating with collagen, laminin, fibrin, and fibronectin for cell migration, proliferation, differentiation, attachment, and promotion for improvement of tissue regeneration, formation, and development. PCL-based materials will develop our understanding of the requirements for developing biological cells, replication, and repair while offering the capacity on different kinds of substrates for some type of tissue implants. Besides, attention to designing multifunctional PCL is on the rise within all healthcare systems and researchers for the suitable alternative and restoration of the soft or hard organ damaged due to trauma, tumors, or any defects.^52,53^ Figure 4 represents an example of an electrospun PCL-based nanofibrous scaffold which is applied in engineering of bone tissue. PCL and its copolymers as biocompatible as well as biodegradable synthetic polymers, have been electrospun into nanofibers and investigated as scaffolds to perform tissue engineering. As mentioned above, polymeric scaffolds such as PCL have employed for cell repairing or help to alter particular gene expression on the transduction of specific transcription factors into the somatic cell for disease modeling and cancer research for application in alternative medicine and tissue engineering. Electrospun nanofibers could be manipulated to regulate the fate of stem cells through determining the appropriate mix of topographic cues, physical properties, and biochemical niches. Different optimizations for electrospinning of PCL Have been described that produced nanofibers in variable parameters. For instance, the smallest diameter of nanofibers reported in the studies is approximately 270 ± 100 nm, including electrospinning a blend of PEG-b-PCL and pure PCL.^54^ Besides, different formulations of electrospinning solutions lead to various outcomes in chemical and, subsequently, biological parameters. For instance, a sheet of nanofibers made of azobenzene functionalized PCL nanofibers exposes a light-responsive difference in wettability.^55^ For another example, a 3D scaffold included in PCL nanofibers and silk fibroin nanoparticles has been assembled to couple the good spatial signs with surface topography and chemistry.^56^ These different optimizations and formulations could help to engine-modified scaffolds according to the target tissues.

Cardiovascular disease, myocardial infarction, and heart muscle problems are relatively common and resulting from heart and vascular system diseases. Disability in patients is associated with the severity of the cardiac defect and reflected the degree of one or more cardiac artery stenosis or cardiac region ischemia. Each year, many reconstructive surgical procedures and invasive bypass surgery are performed to repair heart-tissue defects, traumatic cardiovascular injury, and congenital defect.^58^ The standard care based on electrospun PCL for heart tissue reconstruction is responsible for restoration in injured myocardium cells, leading to increased blood supply in injured myocardium or local angiogenesis. In addition, it has been shown that electrospun PCL–scaffold could increase cardiac progenitor adherence, growth, and human mesenchymal stem cells differentiation into cardiomyocytes.

Moreover, preparation of cardiomyocytes seeded on electrospun PCL scaffolds blend of mesenchymal stem cells is very useful in the initiation phase, regulation the cardiac gene expression such as troponin I and protein production such as myosin heavy chain and alpha-actinin of human cardiac cells and increase the possibility of transplantation of human cells^59,60^ In addition, it has been shown that the arrangement of PCL nanofibers also has a crucial impact on the rate as well as efficiency of differentiation into cardiomyocytes. Safaeijavan et al reported that cardiomyocyte differentiation of AT-MSCs after culture on aligned nanofibrous PCL was considerably increased. At the same time, these cells were cultivated on the random nanofibrous PCL construct.^61^

Spinal cord or nervous system injuries, tendon, or ligament rupture are the most frequent disability that happens in the workplace, accidents, and sports, leading to the body’s subsequent limited movement. Because of the inadequacy of the healing potential of the nerves, the absence of suitable dividing cells and autografts donor site, healing after surgery is slow and insufficient. Incomplete or prolonged repair in the nervous system or new nerve growth is a significant challenge in clinical practice and research. Several scaffolds serve as valuable substrates by incorporating laminin, and nerve growth factor was used to promote the extension of neuritis and enhanced Schwann cell.^52^ In this way, autogenous tendon, tissue, and nervous graft are a gold standard for reconstructing these defects. Yucheng Lin and colleagues investigated the impact of PCL/collagen I (COL-1) on improving regeneration of the tendon and bone interface. They found that PCL/COL-1 hybrid electrospun nanofiber membranes lead to increase tendon-bone healing with increasing supporting cell adherence, growth, and osteogenic separation of the tendon stem/progenitor cells. PCL/COL-1 hybrid membranes are a hopeful scaffold for using tendon-bone tissues in tissue engineering.^62^ Autologous skin grafts, including culturing, expanding, and harvesting in the laboratory, is the most treatment procedures in skin burn and injuries. Collagen/PCL fiber scaffolds cultivated with epidermal keratinocytes of human and dermal fibroblasts supported cellular response, which leads to the generation of the epidermis and dermis with persistent basal cell layers and lower membrane. Furthermore, a study has suggested the application of electrospun collagen nanofibers as a scaffold in the preparation phase of engineered skin to create microenvironments for wound healing. The scaffold permit cell attachment sites, cell–ECM (extracellular matrix) interactions as well as control cell biological activity, differentiation and tissue formation.^63^

PCL-based electrospun nanofibrous scaffolds are not only applicable for multilayer and linear tissues but also could be utilized in bulky organs like bone tissue engineering. An excellent 3D scaffold must be provided structurally like the bulk tissue, alongside physically supporting the healing process of the bone and using biochemical signals to stimulate osteogenesis.^52,64^

In several studies, electrospun PCL nanofibers combined with bioceramics, which were used to improve mechanical characteristics of the nanofibrous scaffold, were used to increase the osteogenic differentiation of stem cells. PCL/chitosan/Zn-doped nHA electrospun nanocomposite,^61^ PCL/ Bio-Oss^ ®,65^ and β‐glycerophosphate loaded PCL/PEO^66^ were demonstrated to have a promising potential to use as a biofunctional and osteoinductive bone-implant while stem cells cultured on them. Following the Chamundeswar and colleagues, a sponge-like 3D nanofiber-based scaffold was developed, a combination of PLA, PCL, and PEO.^67^ This scaffold intended to directly induce the osteogenic differentiation of human MSCs, while avoiding using a medium.^67^ For other instances, a bilayer scaffold assembled by uniaxially aligned PCL fibers coated with a hydrogel made of chitosan and the hyaluronic acid composite has been utilized to develop ligament regeneration.^52,68^

Kidneys are highly complicated organs comprised of various cells. Its complex anatomy and function are some of the most challenging issues in the body to regenerate. Preceding biomedical engineering measures include constructing extracorporeal systems developed to support the renal system, which are made of biological and synthetic elements and external alternatives to the kidney. However, if we can implant such devices for a prolonged time, without using extracorporeal perfusion circuits or medicines that suppress the immune system, patients’ problems would be reduced. Efforts to build an active kidney unit are a successful step in treating kidney failure and are still in progress, and tissue engineering plays an important role. Another way to improve kidney function is to increase kidney tissue with proliferating kidney cells in vitro on an engineered biomaterial that is eventually used for autologous transplantation at the damaged site. Recently, efforts have been made to regenerate renal cells to produce functional nephron units. Hosseini et al developed a PCL scaffold containing bone morphogenetic protein-7 (BMP7) and then monitored the biological behavior of the human embryonic kidney cells (HEK). They showed that the growth rate, spread, and survival of HEK cells increased significantly after culture on PCL/BMP7 nanofibrous scaffolds compared to the empty PCL.^66^

## Electrospun PCL nanofibers and wound healing

As mentioned, electrospinning is a versatile procedure used to generate fibrous scaffolds. Wound healing and wound dressing is one of the leading fields in using electrospun nanofibrous scaffolds and patches. Several polymers (either synthetic or natural) are applied in this regard. PCL is a polymer that can be considered semicrystalline and biodegradable. Hence, PCL is an acceptable vehicle for electrospinning. Lately, PCL-based electrospun nanofibrous constructs are at the center of interest to produce bioactive dressing materials properly to be used for treating wounds, either chronic or acute. PCL is at the top of the list of synthetic polymers applied in electrospun nanofibrous structures engineering for wound dressing purposes. Among the different properties of PCL and its electrospun nanofibrous constructs, some positive features include flexibility, high crystallization rate, long-term durability, and some negative features like steady biodegradation rate, reduced mechanical power, and hydrophobicity were the considered target in wound dressing related studies.^2,69,70^ Furthermore, electrospun PCL nanofibers provide a suitable tool as drug or biological factor delivery systems for use in wound treatment. For instance, PCL nanofiber mats can be embedded with placental-derived bioactive molecules rich in an appropriate matrix for facilitating healing serious full-thickness wounds.^71^

Lately, scholars have focused on developing thoroughly combined, multirole scaffolds capable of providing all the primary characteristics expected for efficient wound dressing and healing process. Remarkable multirole scaffolds are fabricated by combining various biological factors with polymers (either synthetic or natural) for electrospinning. Moreover, multirole scaffolds are forged by combining electrospinning with other high-level methods, including smart mixing of components.^69^ For instance, Golchin et al designed a combined electrospun mesh for a wound dressing that PCL/Carbomer and PVA/Chitosan were electrospun bilaterally. They report that this nanofibrous construct poses considerable capacity to transform the Curcumin and stem cells concurrently and demonstrated the high capacity for usage for wound dressing and skin tissue engineering.^24^ For another instance, Motealleh et al designed the studies to incorporate chamomile in a mix of electrospun PCL and polystyrene for treating wounds.^72,73^ They reported that chamomile was utilized for supporting wound bandage as an active agent for healing wounds.

These modified electrospinning methods have been opened new views for more research and therapeutic application. For instance, Shin et al introduced a radial algorithm by an adapted electrospinning technique to aim that highly coordinated PCL nanofibers can have a better capacity for wound healing combined with different cells.^74^

Inherent mechanical flexibility and weak water vapor permeability are additional appealing properties of PCL for treating wounds. Hence, the authors provided a mix of various features of the elements, PCL, Epidermal growth factor, and PVA/Chitosan, to create a proper blend of multilayer compounds for bandage with improved capacities for healing wounds.^25^ This study demonstrated that this electrospun composite scaffold shows adequate wound closure and enhanced wound healing. Tamayol and colleagues studied the effect of unified electrical heat, PEGylated-chitosan drug carrier loaded PGS−PCL fibrous structure for innovative delivery of medicines using a supple biodegradable wound dressing.^71^ They used PEGylated-chitosan drug carriers into PGS−PCL using electrospinning. The results of this study validated the designing electronically manageable bandage which is capable of carrying biological agents with the on-demanded temporal algorithm.

###  Electrospun PCL nanofibers and drug delivery 

 The limits of standard medications indicate the importance of designing new drugs for the treatment of various diseases. Conventional therapeutics have insufficient circulation time in circulation and do not conform to biological systems. Nanofibers can be regarded as proper drug carriers because of their large surface area and acceptable biocompatibility.

PCL can be considered the primary polymer used in various medical advantages because of its known biocompatibility and biodegradable character in addition to a great variety of degradation properties.^4^ PCL is readily spun into nanofibers through melting or solvent electrospinning. The compatibility of PCL with many drugs leads to the uniform distribution of the ingredients in the matrix. In contrast, long-term delivery of an ingredient by the carrier is a function of slow degradation rate.^75^ Given that the degraded products have no toxicity, the aliphatic polyester of PCL is a leading option for medical applications.^76^ Based on the non-toxic character and approval by the FDA, the application of PCL as an implantable biomaterial and injectable implant for managing the release using delivery systems for drugs is widely studied.^77,78^ PCL contains advantages such as high permeability to small molecules of drugs and compatibility with a large number of medications, which enables uniform distribution of predominantly lipophilic medicines within the carrier matrix because of its hydrophobic property.^36,79^ Drug release duration from PCL substrate delivery systems lasts over one year, even though it can be longer/shorter via appropriate physical or chemical modifications.^80^

**Figure 5:**
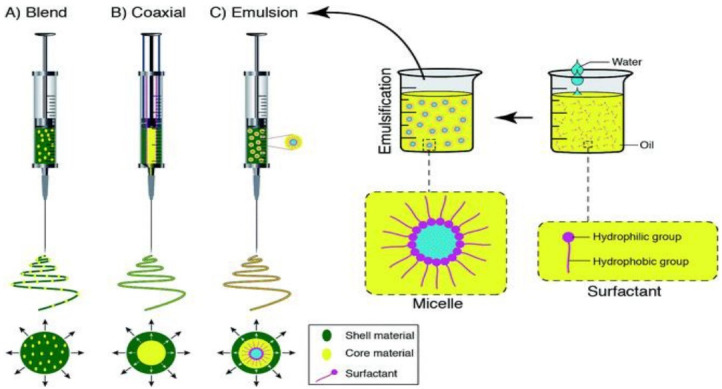
Schematic representations of the spinneret loaded with a bioactive agent for drug delivery system by using (A) blend, (B) coaxial, and (C) emulsion electrospinning. Reprinted with permission from Nikmaram et al,^81^ Royal Society of Chemistry (RSC)

Several types of PCL and delivery systems based on PCL have been defined, including nanoparticles, microparticles, electrospun mats, films, and scaffolds that we focused on electrospun PCL/nanofiber applications in drug delivery. There are different approaches to produce electrospun nanofibers as drug delivery systems; (Figure 5 displays some of these approaches). The unique structure of nanofiber support, recognized by the high surface/contact area, helps a strong drug loading ability and prolonged drug release that leads to malignant cell destruction.^75^ PCL nanofibers are described as drug delivery nanomaterials for cancer therapeutics, antibacterial drugs, non-steroidal anti-inflammatory drugs (NSAIDs), cardiovascular agents, gastrointestinal and contraceptive drugs.

###  Cancer

Electrospun nanofiber scaffolds have superior drug delivery of anticancer therapeutics. In addition to reinforcing the effect and influence of loaded drugs, it reduces unfavorable adverse effects by warranting the highest cellular aggregation of drugs laden.^82^ PCL has applications in the design and development of sustained-release implants due to its hydrophobic and slow eroding nature.^83^ Doxorubicin hydrochloride (DOX) is a crucial and extensively applied ingredient of cancer-related drugs. The micelles encapsulated with DOX have been produced from an FA–PCL–PEG copolymer to function as a strategy to control cancer. Upon implantation close to a solid tumor, fibers start to secret active targeting micelles (which benefit from degradation), followed by accumulation close to tumor tissue.^84^ In 2014, drug delivery systems responsive to pH based on polydopamine-coated PCL nanofibers increased the death rate of cancer cells at lower pH values because of explosive secretion of loaded DOX in vitro.^85^ Likewise, DOX-loaded and pH-responsive core-shell nanofibers have been developed through coaxial electrospinning and withheld high potential for treating cervical cancer.^86^ Similarly, DOX-loaded and core-shell type nanoparticle structures involved in indomethacin (MC) drug-loaded PCL and gelatin fibers have been fabricated for direct application against mouse tumor cells.^87^ The multifunctional system so developed indicated steady release of the drug and inhibition of cancer cells up to 96%, surpassing free DOX. Mellatyar et al developed PCL/PEG nanofibers encapsulated with the 17-DMAG drug having controlled release properties for an extended period of time.^88^ Jain and colleagues reported that administration of piperine-loaded (PCL/Gelatin) nanofibers, as postsurgical implants, was associated with an increased death rate of cancer cells.^89^ Besides, gelatin and PCL fibers loaded with polyaniline nanoparticles have been fabricated to show remarkable inhibition of tumor growth.^90^ Cis-diamminediiodoplatinum nanofiber, a compound used to control delivering drugs, has been embedded into PCL nanofibers, which was successful. It worth noting that the sustained production of cis-DIDP from nanofibers avoids human lung tumor cells in vitro.^91^ In vitro analysis shows that green tea polyphenol (GTP) that is adsorbed on multi-walled carbon nanotubes and loaded on PCL nanofibers is helpful for cancer treatment against human epithelial (A549) and hepatoma (Hep G2) cell lines resulting in anti-proliferative activity against tumor cells.^92^

Curcumin is a natural compound that has significant side effects and a short half-life when injected intravenously. Several studies have reported that curcumin, along with natural extract loaded PCL nanofibers, revealed higher levels of cytotoxicity to fight breast cancer cell line (MCF7) and cancers related to the respiratory system (A459).^93^ Similarly, PEG-PCL polymers incorporate Curcumin as an anticancer agent against Glioma 9L cancer cells.^94^ Yohe and colleagues mentioned the application of air trapped and nanofibers to enhance the stability of PCL electrospun fibers available in hydrophobic polyglycerol monostearate-co-ε-caprolactone (PGC-C18) polymer as well as 2 kinds of anticancer drugs. The fabricated fibers release drugs locally over prolonged periods.^95^

Temozolomide is a well-known chemo drug commonly used to treat patients with various brain tumors such as glioblastoma multiforme and anaplastic astrocytoma. Since this drug is administered orally, it has to travel a long way to reach its destination, which causes the drug to be highly toxic. Therefore, the local release of this drug at the tumor site can significantly increase its effectiveness and reduce its toxicity. A study showed that incorporating the Temozolomide in the PCL nanofibrous scaffold can substantially induce apoptosis to the U87 glioma cells. At the same time, its initial burst release was also decreased, which is a good characteristic of this composite.^96^

To overcome the challenge of the side effects of chemical drugs in treating different cancers, the application of exosomes extracted from stem cells has raised great hopes today, considering that it does not have the problems caused by stem cell transplantation, such as transplant rejection and tissue incompatibility. The exosomes extracted from adipose tissue-derived mesenchymal stem cells (AT-MSCs) are extensively studied in recent years. Fortunately, it has been shown that AT-MSCs derived exosomes can be well embedded in the PCL nanofibrous scaffolds and induce apoptosis in breast cancer cells (MCF7).^97^ In contrast, not only did they not reduce the growth and proliferation of normal cells, they even played a supportive role in their growth and proliferation.^97^

###  Antimicrobial

 The smart antibiotic delivery system extensively uses the electrospun nanofiber scaffolds to assess the production of drugs after induction by biological factors such as pH, temperature, and UV-light sensitivity.

It has been reported that electrospun PCL/poly(trimethylene carbonate) (PTMC) ultrafine composite fiber mats function as drug-delivering materials encapsulating herbal antibacterial agents and are efficiently applied to treat skin bacterial infections or wound healing.^98^

Chitosan /PCL/ciprofloxacin HCl was investigated for its release features as well as antimicrobial activity. Besides, Nnanofibers containing PCL and ampicillin were produced and tested versus *Staphylococcus aureus* and* Klebsiella pneumonia*. It was revealed to release burst within the first hour, which was completed in the next 96 hours.^99^ Continuous secretion of metronidazole benzoate (MET) by PCL nanofibers has been found to depend on the solvent ratio and drug concentration.^100^ Similarly, Salicylic acid and PCL/ PEG having a cross-linked PEG surface were developed, showing a common biphasic appliance for release along with steady release rates that are controlled by the thickness of PEG shell in a linear correlation.^101^

###  Non-steroidal anti-inflammatory drugs 

NSAIDs and steroids are not similar, steroids are famous worldwide because of anti-inflammatory and reduction of the synthesis of prostaglandins. Also, NSAIDs are generally prescribed because of their known pain-relieving, anti-pyretic, as well as blood clotting functions. Nevertheless, NSAIDs also have negative consequences. The prevalence of weak water-soluble NSAIDs is on the rise in the pharmacy firms. To reach an acceptable goal to improve treating NSAIDs, electrospinning methods are previously used in this industry. Ibuprofen is a NSAID commonly prescribed for sedating ache, fever, swell, migraine, joint diseases, and hurting menstruation. Naproxen sodium is a cyclooxygenase inhibitor that belongs to the NSAIDs category, which prescribes inflammation treatment. Recently, Naproxen and its salt (naproxen sodium) are electrospun with PCL polymers having perfect drug loading capacity to achieve quick action, which circumvents fast hepatic metabolism and becomes easily accessible via sublingual administration.^102^ Recently, one study indicated that PCL nanofibers loaded with ibuprofen could augment the release rate of this medicine in a biomedical context in which more than 99% of the drug was released from the fabricated nanofibers in 4 hours.^103^ A cardiovascular drug such as carvedilol binds to and inhibits alpha and beta-adrenergic receptors for healing congestive heart failure. Potrč and colleagues investigated electrospun scaffolds of PCL nanofiber as the delivery method for oral administration of weakly water-soluble carvedilol. It has been reported PCL electrospun nanofibers contribute about 76% of carvedilol release only in 4 hours, indicating a considerable enhancement of the release rate of these weakly water-solvable drugs.^104^

###  Gastrointestinal drugs

Gastrointestinal drugs are antidiarrheal, antiemetic, and anti-ulcer agents, as well as cathartics regulating gut motility, water flow, and improving the digestion in patients.^105^ Jaber et al have recently developed a core/shell nanofiber by PVA/PCL to load metoclopramide hydrochloride as a gastrointestinal drug carriers to reach a manageable release pattern protecting sensitive ingredients in biological pH.^106^

###  Contraceptives

Long-term and manageable release of contraceptive steroids in injectable and embedded forms is favorable for sustained treatment, patient compliance as well as simple, clinically expense savings, and reducing invasive procedures. Levonorgestrel is a drug with hydrophobic properties following a zero-order release mechanism from PCL matrices. The release period could be expanded by changing the weight of the polymer, particle dimension, and concentration. As a subdermal PCL contraceptive implant, Capronor is designed for the long carry of levonorgestrel. In Phases I and II, pharmacological findings were acceptable.^107^

## Conclusion and Outlook

Due to the convenience and viable setup of electrospinning parameters, great attention has been paid to developing wide-ranging nanofibers in different uses, especially biomedicine. The materials utilized for fabricating scaffolds must be non-cytotoxic, biocompatible, and bioabsorbable/biodegradable. Hence, several biopolymers have been familiarized, such as PCL, PLA, PVA, Chitosan, etc., that satisfying the above rules are assuring materials. According to our study, PCL makes a potential candidate biopolymer that can be combined with other materials forming copolymers and composites with the primary mechanical and physicochemical properties as per the particular demands. Based on our literature review, electrospun PCL nanofibrous compounds have shown progress in mechanical characteristics that are important for biological strategies, especially in regenerative medicine. PCL has accessible manipulation routes among various mixed polymers and can be administrated to various forms and magnitudes because of low melting temperature and excellent viscoelastic features. These features, alongside other supreme properties of PCL, include prominent mechanical characteristics, compatibility with other polymers, and biodegradability, making it one of the attractive polymers for biomedical applications. Among the available scaffold engineering methods, electrospinning is an efficient, simple, and cost-effective technique based on electro-hydrodynamic systems depending on an electrified viscous fluid jet drawn by the air toward a collector at varying electric potency. Recently, PCL-based biomaterial scaffolds were developed via different procedures, mixing particular advantages of the 3D printing method and electrospinning. The various numbers of polymers and biological factors combined by PCL, alongside the distinct advantages of electrospinning-based methods, have attracted interest and displayed high potency in biomedical uses. Prolonged, controlled drug release in PCL and its copolymers electrospun nanofibrous composites is beneficial for sustained therapy, cost-effective, reduced invasive surgical operations, and patient agreement. As we know, scaffolds are one of the critical requirements of tissue engineering. The finely designed electrospun scaffolds could be utilized for directing the differentiation of stem cells in 3D structures that mimic in vivo microenvironments. Literature reviews demonstrated that electrospun PCL-based nanofibrous scaffolds offer accepted architectures at the nano to micro-scale with desired porosity for selective movement of small molecules and cells, forming a suitable 3D matrix for engineering molds. Therefore, electrospun PCL-based nanofibers have confirmed their suitability for developing promising platforms for tissue engineering and administration relative to regenerative therapies. Significant progress is achieved during the past years in the fabrication of electrospun PCL and its copolymers nanofibrous composites in biomedical applications. Additional studies are required to increase translation of these products for clinical application in drug-carrying means, tissue-engineered scaffolds, and other biomedical-based composites. Hence, there are still several challenges that need to be defeated in future studies, especially in reducing problems and limitations of PCL, such as hydrophobicity and low cell adhesion. It is recommended that the procedure of electrospinning be administered correctly in well-ventilated fume hoods with the best functional requirements, and its setup for the different conditions should be sufficiently studied. The continuous progress provides valuable information regarding recent progress and problems of PCL and its copolymers electrospun nanofibrous composites as an essential tool of new biomedical-based procedures for various applications. As mentioned above, PCL nanofibers mimic the ECM; its capacity for cell attachment and proliferation has been restricted to hydrophobic properties. Hence, different studies present some suggestions to overcome this disadvantage, such as high porosity on the exterior of the PCL nanofibers and copolymerization with other hydrophilic polymers. Modified electrospinning methods are other options that can improve the features of PCL in nanofibrous scaffolds. Summary, electrospun PCL nanofibers are known to be hopeful for biomedical uses thanks to their special valuable features. The overall results suggest that PCL and its copolymer electrospun nanocomposite scaffolds may potentially have extensive use in biomedical applications soon.

 To sum up, electrospun PCL-based nanofibrous structures have achieved notable success in some biomedical branches, such as drug delivery systems of cancer treatment and bone tissue engineering. However, more effort is needed to continue evaluating more efficient structures. Furthermore, the novel combining techniques of electrospinning should be further investigated. However, with overcoming some challenges, electrospun PCL nanofibrous structure will remain a promising construction for developing biomedical applications.

## Ethical Issues

 Not applicable.

## Conflict of Interest

 The authors declare that they have no conflicts of interest.
